# Alteration of choroidal thickness in a case of carotid cavernous fistula: a case report and a review of the literature

**DOI:** 10.1186/1471-2415-13-75

**Published:** 2013-12-05

**Authors:** Yoichiro Shinohara, Tomoyuki Kashima, Hideo Akiyama, Shoji Kishi

**Affiliations:** 1 Department of Ophthalmology, Gunma University School of Medicine, 3-39-15 Showamachi, Maebashi, Gunma 371-8511, Japan

## Abstract

**Background:**

To measure the alterations of the choroidal thickness in Carotid cavernous fistula (CCF) using enhanced depth imaging optical coherence tomography (EDI-OCT).

**Case presentation:**

A 64-year-old woman was referred to us for redness, exophthalmos and visual disturbance in her right eye. She was diagnosed with CCF by magnetic resonance imaging (MRI) and magnetic resonance angiography.

Observations; Embolization resulted in improvement of ocular symptoms, and there was a reduction of the subfoveal choroidal thickness in the right eye from 351 μm preoperatively to 142 μm postoperatively in EDI-OCT.

**Conclusion:**

EDI-OCT demonstrated that the choroidal thickness increases occurred due to congestion in a CCF case.

## Background

Carotid cavernous fistula (CCF) is an abnormal connection between the carotid arterial system and the cavernous sinus [1]. These cases typically have a low flow and present with oculo-orbital venous congestive features such as exophthalmos, chemosis, and sometimes oculomotor or abducens cranial nerve palsy [2]. Angiography is an essential tool for making a direct diagnosis and therapeutic determination of CCF. Arteriovenous fistula occurs after trauma or develops spontaneously after rupture of a cavernous carotid aneurysm or a preexisting weakness of the arterial wall.

Barrow et al. have classified CCF into four types based on the arterial system involved. Type A, or direct, CCF is high-flow shunts between the main trunk of internal carotid artery and the cavernous sinus. The other three types are indirect, or dural, CCFs, which arise from the dural branches of internal, external carotid system or both (type B, C, and D) [3]. Even though some dural fistulas close spontaneously, therapeutic intervention of dural fistulas should be reserved for the patients with visual deterioration. Intravascular coil occlusion of the superior and inferior ophthalmic veins and cavernous sinus of the symptomatic eye is a highly efficient and safe treatment for these conditions [4,5]. Previous studies have demonstrated that one of the typical symptoms of CCF is congestion of the ocular veins, such as the conjunctival, episcleral and retinal veins. However, dural arteriovenous fistula sometimes exhibits a milder shunt flow and symptoms [2,6-8]. In the current report, we used enhanced depth imaging optical coherence tomography (EDI-OCT) (Spectralis; Heidelberg Engineering, Heidelberg, Germany) before and after an endovascular embolization and documented the alterations of the choroidal thickness in a CCF patient.

## Case presentation

**Figure 1:**
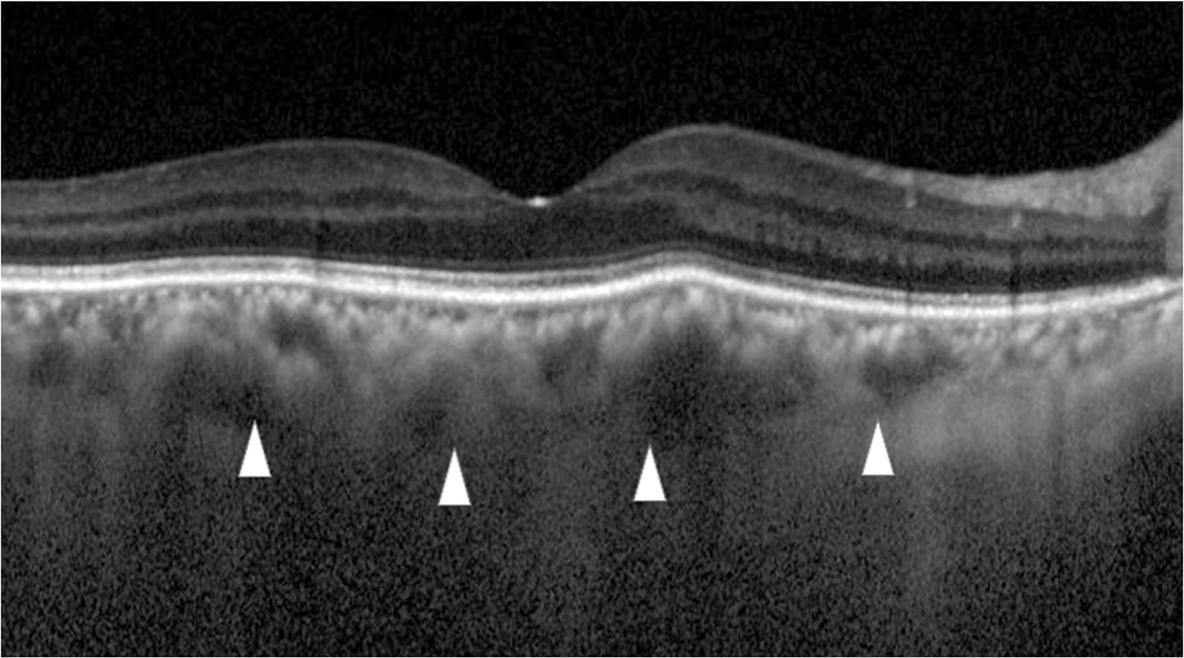
**Left photograph: prior to treatment, the episcleral and conjunctival vessels exhibit tortuosity and dilatation.** Right photograph: After treatment, the vessels returned to normal.

**Figure 2:**
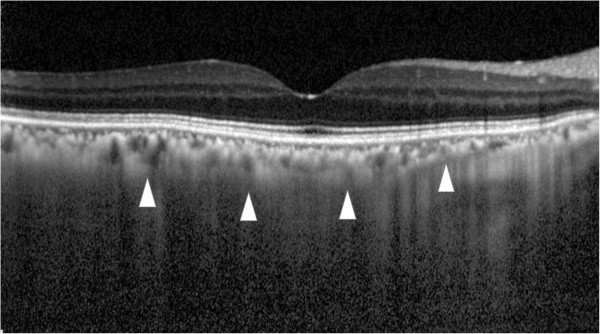
**Right eye fundus photographs.** Left photograph: Prior to treatment, the retinal veins exhibit dilatation and tortuosity due to CCF. Right photograph: After treatment, the venous condition returned to normal.

**Figure 3:**
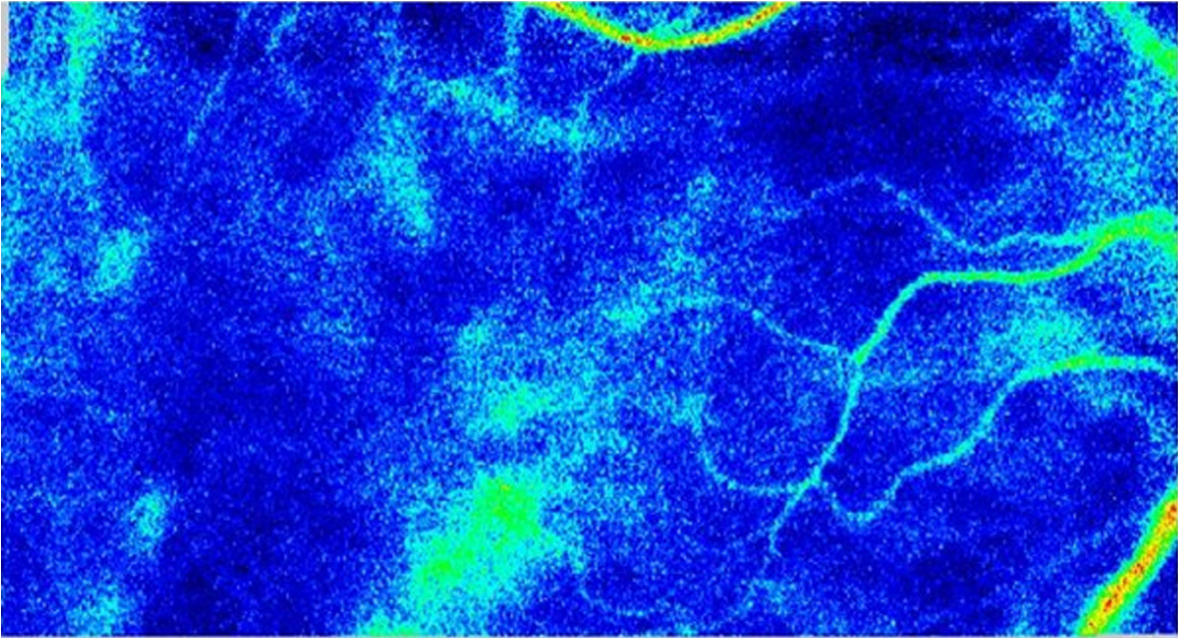
**Enhanced depth imaging optical coherence tomography (EDI-OCT) before and after treatment.** Upper image: Prior to treatment, the subfoveal choroidal thickness is 297 μm (arrowheads). Lower image: After treatment, the subfoveal choroidal thickness decreased to 142 μm, which is almost half of the preoperative choroidal thickness (arrowheads).

**Figure 4:**
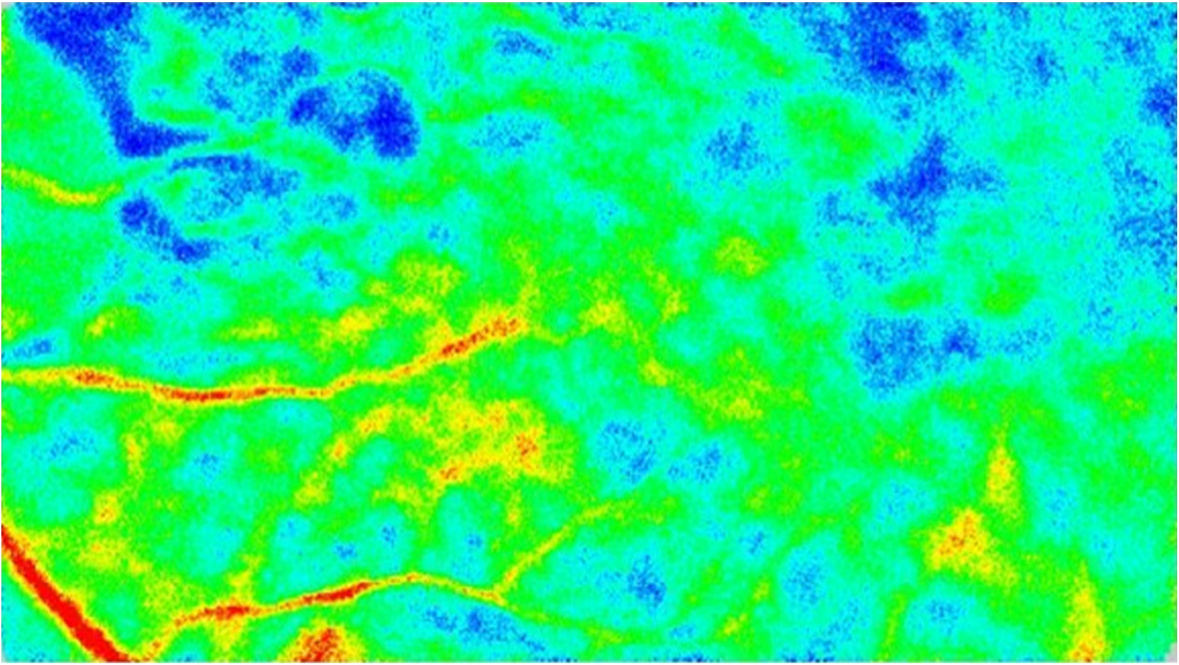
**Preoperative blood flow image obtained by laser speckle flowgraphy Upper fundus image shows the right eye.** The fundus color that is primarily seen shows that the choroidal blood flow is cold, which indicates there is a decrease in the choroidal blood speed due to venous congestion. Lower fundus image shows the left eye. In this image, the color is warmer as compared to that observed in the right fundus image.

A 64-year-old woman developed redness and exophthalmos of her right eye. After undergoing magnetic resonance imaging (MRI), her family doctors, including an ophthalmologist and radiologist, previously diagnosed Graves’ disease based on extraocular muscle swelling and the lack of CCF evidence in the MRI. Although the patient underwent intravenous/oral steroid therapy and radiation therapy treatments, her ocular symptoms did not improve for four months. Since she began to gradually exhibit visual disturbance in her right eye, she was subsequently referred to our hospital 4 month after her symptom. At her first hospital visit, the best-corrected visual acuity of her right and left eye was 20/60 and 20/13, respectively. Intraocular pressure was 15 mmHg in both eyes. Right eye had 3 mm greater protrusion than left eye. (Right = 20 mm Left = 17 mm) Even though her ocular and systemic examinations did not show any signs of thyroid ophthalmopathy except for exophthalmos, we observed dilatation, tortuosity and venous congestion of the conjunctiva and retinal vein in her right eye (Figures 1 and 2). Optic nerve edema was not observed. Laboratory tests, which included thyroid profiles such as T3, T4, TSH and TSH receptor antibody, were all within the normal ranges. MRI and magnetic resonance angiography (MRA) determined there was a low flow, which led to suspicion of cavernous sinus-dural arteriovenous fistula (CS-DAVF). Then we performed cerebral angiography and confirmed CS-DAVF which was located at right supraposterior carotid to right superior orbital vein sinus supplied by branch from bilateral middle meningeal artery and meningohypophyseal trunk. The result also revealed subsequent congestion of the choroid in her right eye. One week after the angiography, we performed embolization. Three months after the surgery, embolization of the fistula relieved both the tortuosity and dilatation of the conjunctiva or retinal vein in her right eye. Repeated angiography showed no existence of CS-DAVF and choroidal congestion. Best-corrected visual acuity in her right eye improved to 20/13, all the ophthalmologic signals and symptoms had disappeared. Postoperative MRI showed the dilatation of the superior ophthalmic vein had disappeared. EDI-OCT indicated there was a reduction of the subfoveal choroidal thickness in her right eye from 351 μm preoperatively to 142 μm postoperatively (Figure 3). Laser speckle flowgraphy (LSFG) results showed that the fundus image in her right eye was represented with a cold color as compared to that for her left eye (Figure 4), which indicated congestion of the choroidal blood flow in the right eye. Because LSFG usually shows variation of choroidal blood blow due to the change of systemic condition, calculated affected right eye/ normal left eye ratio showed 55% increase of choroidal blood volume after the operation(before 36.4% after 56.3%). All the clinical surveillance and treatments performed in this patient and during the study complied with the Tenets of the Declaration of Helsinki. Written informed consent was obtained from the patient for publication of this case report and any accompanying images. A copy of the written consent is available for review by the Editor of this journal.

## Conclusions

The newly developed EDI-OCT technique has enabled visualization of the choroid. Moreover, use of this method has made it possible to measure the choroidal thickness without having to perform an invasive examination [9,10]. EDI-OCT examinations of eyes with central serous choroidopathy have reported finding a significantly thicker choroidal thickness as compared to that observed in normal eyes [11,12]. Furthermore, EDI-OCT has shown that the choroid is thinner in cases of myopic shift or axial length elongation [13]. Due to the recent progress that has been made when using new imaging techniques, such as EDI-OCT and swept source OCT, our current study decided to focus on the choroid. Since indocyanine green angiography has been shown to be able to detect CCF [14], we were able to diagnose CCF in our current case. Upon further examination with EDI-OCT and cerebral angiography, we could subsequently prove that the choroidal thickness increases occurred due to congestion. Since we also determined that the postoperative choroidal thickness was significantly thinner than normal, this suggests the change may be due to the choroidal atrophy that was caused by the long-standing congestion. Although the preoperative blood flow was shown to be slow due to congestion, LSFG which commonly express choroidal blood flow volume [15] demonstrated there was drastic increase in the postoperative blood flow volume nevertheless the volume was not recovered as high as left eye because longstanding congestion may lead choroidal atrophic change. To the best of our knowledge, this is the first report that has proved the relationship between an increased choroidal thickness by EDI-OCT and choroidal congestion by cerebral angiography in CS-DAVF.

## Competing interests

The authors declare that they have no competing interests.

## Authors’ contributions

AH, SK participated in the design of the study and performed the statistical analysis. YS, TK conceived of the study and participated in its design and coordination and helped to draft the manuscript. All authors read and approved the final manuscript.

## Pre-publication history

The pre-publication history for this paper can be accessed here:

http://www.biomedcentral.com/1471-2415/13/75/prepub
